# Supplementary material for: TP53 Co-Mutation Status Association with Clinical Outcomes in Patients with EGFR-Mutant Non-Small Cell Lung Cancer
Source: Cancers (Basel). 2022 Dec 12;14(24):6127. doi: 10.3390/cancers14246127 (PMC9776757; doi:10.3390/cancers14246127)
Supplement: Supplementary file 1 [file cancers-14-06127-s001.zip › cancers-1978137-supplementary.pdf]

cancers

MDPI

Supplementary Materials

Table S1. Treatment Patterns in 1L and 2L – By Drugs.

1L				2L			
**Therapy**	**Overall**	***TP53wt***	***TP53mt***	**Therapy**	**Overall**	***TP53wt***	***TP53mt***
	**n = 356**	**n = 146**	**n = 210**		**n = 169**	**n = 61**	**n = 108**
Osimertinib	154 (43.3)	63 (43.2)	91 (43.3)	Osimertinib	81 (47.9)	30 (49.2)	51 (47.2)
Erlotinib	65 (18.3)	25 (17.1)	40 (19.0)	Erlotinib	15 (8.9)	6 (9.8)	9 (8.3)
Afatinib	44 (12.4)	18 (12.3)	26 (12.4)	Afatinib	18 (8.3)	3 (4.9)	11 (10.2)
Carboplatin + Pemetrexed	18 (5.1)	11 (7.5)	7 (3.3)	Carboplatin + Pembrolizumab + Pemetrexed	7 (4.1)	2 (3.3)	5 (4.6)
Carboplatin + Pembrolizumab + Pemetrexed	16 (4.5)	5 (3.5)	11 (5.2)	Carboplatin + Pemetrexed	6 (3.6)	2 (3.3)	4 (3.7)
Carboplatin + Paclitaxel	12 (3.4)	4. (2.7)	8 (3.8)	Nivolumab	6 (3.6)	5 (8.2)	1 (0.9)
Clinical Study Drug	8 (2.2)	3 (2.1)	5 (2.4)	Clinical Study Drug	5 (3.0)	2 (3.3)	3 (2.8)
Bevacizumab + Carboplatin + Pemetrexed	5 (1.4)	2 (1.4)	3 (1.4)	Gefitinib	4 (2.4)	4 (6.6)	0 (0)
Gefitinib	4 (1.1)	1 (0.7)	3 (1.4)	Atezolizumab + Bevacizumab + Carboplatin + Paclitaxel	3 (1.8)	2 (3.3)	1 (0.9)
Bevacizumab + Erlotinib	3 (0.8)	1 (0.7)	2 (1.0)	Carboplatin + Osimertinib + Pembrolizumab + Pemetrexed	3 (1.8)	0 (0)	3 (2.8)
Bevacizumab + Carboplatin + Paclitaxel	2 (0.6)	1 (0.7)	1 (0.5)	Bevacizumab + Carboplatin + Pemetrexed	2 (1.2)	0 (0)	1 (0.9)
Bevacizumab + Osimertinib	2 (0.6)	1 (0.7)	1 (0.5)	Bevacizumab + Erlotinib	2 (1.2)	1 (1.6)	1 (0.9)
Carboplatin + Paclitaxel + Pembrolizumab	2 (0.6)	1 (0.7)	1 (0.5)	Clinical Study Drug + Erlotinib	2 (1.2)	2 (3.3)	0 (0)
Pembrolizumab	2 (0.6)	2 (1.4)	0 (0)	Afatinib + Nivolumab	1 (0.6)	0 (0)	1 (0.9)
Bevacizumab + Carboplatin + Erlotinib + Pemetrexed	1 (0.3)	0 (0)	1 (0.5)	Atezolizumab + Bevacizumab-Awwb + Carboplatin + Paclitaxel	1 (0.6)	0 (0)	1 (0.9)
Bevacizumab + Carboplatin + Paclitaxel Protein-Bound	1 (0.3)	0 (0)	1 (0.5)	Atezolizumab + Bevacizumab-Bvzr + Carboplatin + Paclitaxel	1 (0.6)	0 (0)	1 (0.9)

Bevacizumab-Awwb + Osimertinib	1 (0.3)	0 (0)	1 (0.5)	Atezolizumab + Bevacizumab-Bvzr + Carboplatin + Paclitaxel Protein-Bound	1 (0.6)	0 (0)	1 (0.9)
Carboplatin + Cyclophosphamide + Pembrolizumab + Pemetrexed	1 (0.3)	0 (0)	1 (0.5)	Bevacizumab + Carboplatin + Osimertinib + Paclitaxel	1 (0.6)	0 (0)	1 (0.9)
Carboplatin + Docetaxel	1 (0.3)	0 (0)	1 (0.5)	Bevacizumab-Awwb + Cabozantinib + Osimertinib	1 (0.6)	0 (0)	1 (0.9)
Carboplatin + Erlotinib + Paclitaxel	1 (0.3)	0 (0)	1 (0.5)	Bevacizumab-Awwb + Pemetrexed	1 (0.6)	0 (0)	1 (0.9)
Carboplatin + Erlotinib + Paclitaxel Protein-Bound	1 (0.3)	1 (0.7)	0 (0)	Carboplatin + Clinical Study Drug + Pemetrexed	1 (0.6)	0 (0)	1 (0.9)
Carboplatin + Osimertinib + Pembrolizumab + Pemetrexed	1 (0.3)	1 (0.7)	0 (0)	Carboplatin + Erlotinib + Pembrolizumab + Pemetrexed	1 (0.6)	0 (0)	1 (0.9)
Carboplatin + Osimertinib + Pemetrexed	1 (0.3)	0 (0)	1 (0.5)	Carboplatin + Osimertinib + Pemetrexed	1 (0.6)	1 (1.6)	0 (0)
Carboplatin + Paclitaxel Protein-Bound	1 (0.3)	0 (0)	1 (0.5)	Carboplatin + Paclitaxel	1 (0.6)	0 (0)	1 (0.9)
Carboplatin + Paclitaxel + Pemetrexed	1 (0.3)	1 (0.7)	0 (0)	Clinical Study Drug + Osimertinib	1 (0.6)	0 (0)	1 (0.9)
Carboplatin + Pembrolizumab	1 (0.3)	1 (0.7)	0 (0)	Erlotinib + Pembrolizumab	1 (0.6)	0 (0)	1 (0.9)
Cisplatin + Etoposide	1 (0.3)	0 (0)	1 (0.5)	Everolimus	1 (0.6)	0 (0)	1 (0.9)
Cisplatin + Pemetrexed	1 (0.3)	1 (0.7)	0 (0)	Fulvestrant	1 (0.6)	0 (0)	1 (0.9)
Clinical Study Drug + Osimertinib	1 (0.3)	1 (0.7)	0 (0)	Gemcitabine + Osimertinib	1 (0.6)	1 (1.6)	0 (0)
Erlotinib + Pembrolizumab	1 (0.3)	0 (0)	1 (0.5)	Olaparib	1 (0.6)	0 (0)	1 (0.9)
Gemcitabine	1 (0.3)	1 (0.7)	0 (0)	Osimertinib + Pembrolizumab	1 (0.6)	0 (0)	1 (0.9)
Methotrexate	1 (0.3)	1 (0.7)	0 (0)	Pembrolizumab	1 (0.6)	0 (0)	1 (0.9)
Nivolumab	1 (0.3)	0 (0)	1 (0.5)				

All data are presented as n (%).

1L = first-line; 2L = second-line; EGFR-TKI = epidermal growth factor receptor-tyrosine kinase inhibitor; Other = clinical study drug; *TP53mt* = *TP53* co-mutation; *TP53wt* = *TP53* wild-type; VEGF = vascular endothelial growth factor

EGFR mutation includes both exon 19 and exon 21 mutations.

Table S2. Univariate Analysis–rwORR and rwDCR.

Variable	Category	Tumor response, OR (95%CI)	
		rwORR	rwDCR
Overall			
Age at adv dx	≥75 vs. <75	0.7 (0.4-1.3)	0.7 (0.3-1.7)
Sex	Male vs. Female	0.8 (0.4-1.4)	0.5 (0.2-1.2)
ECOG	1+ vs. 0	0.9 (0.5-1.9)	0.6 (0.2-1.9)
	Unknown vs. 0	0.7 (0.3-1.4)	0.5 (0.1-1.6)
Smoking history	Yes vs. No	0.5 (0.3-0.9) [a]	0.5 (0.2-1.2)
TP53	mt vs. wt	0.6 (0.3-1.1)	0.6 (0.2-1.6)
TP53 status/ EGFR mutation type	*TP53*mt/Exon19 vs *TP53*wt/Exon19	0.7 (0.3-1.6)	0.7 (0.2-2.3)
	*TP53*mt/Exon21 vs *TP53*wt/Exon19	0.4 (0.2-1.0) [a]	0.7 (0.2-2.6)
	*TP53*wt/Exon21 vs *TP53*wt/Exon19	0.8 (0.3-2.2)	1.3 (0.2-7.6)
Bone mets	Yes vs. No	1.2 (0.6-2.4)	2.5 (0.7-8.8)
Brain mets	Yes vs. No	1.9 (0.9-4.0)	1.8 (0.5-6.4)
Exon group	Exon21 vs. Exon19	0.6 (0.4-1.2)	1.1 (0.4-2.7)
No TKI Monotherapy			
Age at adv dx	≥75 vs. <75	0.3 (0.1-2.0)	0 (0-8.1)
Sex	Male vs. Female	0.6 (0.1-3.3)	1.0 (0.1-12.3)
ECOG	1+ vs. 0	3.4 (0.4-38.0)	0.7 (0.0-13.0)
	Unknown vs. 0	1.0 (0.1-7.1)	0.5 (0.0-8.6)
Smoking history	Yes vs. No	0.2 (0.0-1.0) [a]	0.2 (0.0-3.1)
TP53	mt vs. wt	1.7 (0.3-9.0)	1.0 (0.1-12.3)
TP53 status/ *EGFR* mutation type	*TP53m*t/Exon19 vs *TP53wt*/Exon19	2.0 (0.2-18.3)	2.8 (0.1-55.2)
	TP53mt/Exon21 vs *TP53wt*/Exon19	7.3 (0.5-111.1)	2.8 (0.1-55.2)
	TP53wt/Exon21 vs *TP53wt*/Exon19	4.0 (0.3-64.0)	69577.7 (0.0-NA)
Bone mets	Yes vs. No	1.6 (0.2-15.6)	0.4 (0.0-5.7)
Brain mets	Yes vs. No	0.5 (0.1-3.5)	0.1 (0.0-1.2)
Exon group	Exon21 vs. Exon19	3.5 (0.6-21.4)	2.4 (0.2-29.1)
TKI Monotherapy			
Age at adv dx	≥75 vs. <75	0.8 (0.4-1.4)	1.2 (0.4-3.4)
Sex	Male vs. Female	0.8 (0.4-1.5)	0.4 (0.2-1.2)
ECOG	1+ vs. 0	0.8 (0.4-1.7)	0.5 (0.1-2.0)

	Unknown vs. 0	0.6 (0.3-1.3)	0.5 (0.1-1.8)
Smoking history	Yes vs. No	0.6 (0.3-1.1)	0.5 (0.2-1.4)
TP53	mt vs. wt	0.5 (0.3-1.0)	0.6 (0.2-1.7)
TP53 status/ *EGFR* mutation type	*TP53*mt/Exon19 vs *TP53*wt/Exon19	0.6 (0.2-1.5)	0.5 (0.1-2.1)
	*TP53*mt/Exon21 vs *TP53*wt/Exon19	0.3 (0.1-0.7) [b]	0.5 (0.1-2.4)
	*TP53*wt/Exon21 vs *TP53*wt/Exon19	0.6 (0.2-1.9)	0.9 (0.1-5.6)
Bone mets	Yes vs. No	1.2 (0.6-2.4)	3.6 (0.8-16.2)
Brain mets	Yes vs. No	2.3 (1.0-5.4)	5.5 (0.7-42.3)
Exon group	Exon21 vs. Exon19	0.5 (0.3-0.9) [a]	1.0 (0.4-2.6)

[a] $p \leq 0.05$; [b] $p \leq 0.01$; [c] $p \leq 0.001$; [d] $p \leq 0.0001$

rwDCR = real-world disease control rate; ECOG PS = Eastern Cooperative Oncology Group performance status; EGFR = epidermal growth factor receptor; mets = metastases; NSCLC = non-small cell lung cancer; rwORR = real-world overall response rate; *TP53*mt = *TP53* co-mutation; *TP53*wt = *TP53* wild-type

Cox analysis for time-to-event outcomes, and logistic regression analysis for tumor response.

Other monotherapy: non-TKI monotherapies

exon 19 = exon 19 deletions, and exon 21 = exon 21L858R mutations

Table S3. rwORR, rwDCR, and rwBOR – *TP53*mt vs. *TP53*wt.

**Variable**	**Overall**	***TP53wt***	***TP53mt***	***p* -value**
	**n = 275**	**n = 106**	**n = 169**	
**rwORR, %**	78.0 (73.0-83.0)	83.0 (75.0-90.0)	75.0 (68.0-81.0)	0.1240
rwDCR, %	92.0 (89.0-95.0)	94.0 (89.0-98.0)	91.0 (86.0-95.0)	0.3285
rwBOR Category, n (%)				
CR	20 (6.9)	12 (10.8)	8 (4.5)	-
PR	206 (71.0)	80 (72.1)	126 (70.4)	-
Stable disease	42 (14.5)	13 (11.7)	29 (16.2)	-
PD	22 (7.6)	6 (5.4)	16 (8.9)	-
rwDoR in months, median (range)	n = 206	n = 122	n = 84	
	9.5 (7.9-11.9)	11.9 (8.4-15.5)	9.0 (6.9-11.1)	0.5403

All data are presented as % (95% CI), unless specified. Tumor response sample = 275 (*TP53*wt: 106; *TP53*mt = 169) .CR = complete response; DoR = duration of response; n = no. of patients; PR = partial response; PD = progressive disease; rwBOR = real-world best overall response; rwORR = real-world overall response rate; rwTR = real-world tumor response; SD = standard deviation; *TP53*mt = *TP53* co-mutation; *TP53*wt = *TP53* wild-type.
EGFR mutation includes both exon 19 and exon 21 mutations.

Table S4. Efficacy Outcomes – *EGFR*-TKI Monotherapy.

Variable	Overall	*TP53wt*	*TP53mt*	HR (95% CI)	*p* -value
rwORR, %	n = 239	n = 94	n = 145		
	77.0 (72.0-83.0)	84.0 (76.0-91.0)	73.0 (66.0-80.0)	NA	0.0624
rwDCR, %					
	92.0 (89.0-9.05)	94.0 (90.0-99.0)	91.0 (86.0-95.0)	NA	0.2967
	n = 267	n = 107	n = 160		
Median rwPFS, months (95% CI)	14.0 (11.3-15.6)	16.4 (12.7-21.2)	12.0 (9.0-14.1)	1.6 (1.1-2.3)	0.0069
rwPFS Rate at 24 months, % (95% CI)	26.0 (19.0-33.0)	34.0 (22.0-47.0)	20.0 (12.0-29.0)		-
	n = 267	n = 107	n = 160		
Median OS, months (95% CI)	29.2 (25.5-36.6)	44.7 (28.7-NA)	25.3 (22.4-30.2)	1.7 (1.1-2.6)	0.0121
OS Rate at 24 months, % (95% CI)	62.0 (55.0-69.0)	71.0 (60.0-80.0)	56.0 (46.0-65.0)		-
rwDoR in months, median (range)	n = 173	n = 73	n = 100		
	6.9 (3.2-11.3)	7.2 (3.2-10.8)	6.1 (3.2-11.6)	NA	0.5496

CI = confidence interval; mo = months; n = no. of patients; rwDoR = duration of response; rwORR = real-world overall response rate; OS = overall survival; rwPFS = real-world progression-free survival; *TP53mt* = *TP53* co-mutation; *TP53wt* = *TP53* wild-type

EGFR mutation includes both exon 19 and exon 21 mutations.

Table S5. Efficacy Outcomes – By *EGFR*-TKI Generation.

Variable	Overall	*TP53wt*	*TP53mt*	HR (95% CI)	*p* -value
By EGFR-TKI Generation					
**1st Generation**					
rwORR, %	**n = 63**	**n = 23**	**n = 40**		
	74.0 (63.0-85.0)	65.0 (45.0-84.0)	80.0 (67.0-92.0)	NA	0.1944
rwDCR, %	**n = 63**	**n = 23**	**n = 40**		
	87.0 (79.0-95.0)	86.0 (73.0-100.0)	87.0 (77.0-97.0)	NA	0.9503
rwDoR in months, median (range)	**n = 43**	**n = 15**	**n = 28**		
	7.4 (4.1-9.5)	7.7 (4.1-9.1)	5.9 (4.1-10.9)	NA	0.9295
	**n = 69**	**n = 26**	**n = 43**		
Median rwPFS, months (95% CI)	9.9 (8.2-12.0)	11.3 (9.1-22.5)	9.0 (6.0-10.9)	1.9 (1.0-3.5)	0.0399
rwPFS Rate at 24 months, % (95% CI)	14.0 (7.0-25.0)	27.0 (10.0-48.0)	8.0 (2.0-20.0)	NA	NA
	**n = 69**	**n = 26**	**n = 43**		
Median OS, months (95% CI)	29.2 (25.1-38.2)	42.0 (25.5-NA)	27.6 (16.1-33.2)	1.6 (0.9-3.0)	0.1378
OS Rate at 24 months, % (95% CI)	65.0 (52.0-75.0)	82.0 (59.0-92.0)	56.0 (39.0-69.0)	NA	NA
**2nd Generation**					
rwORR, %	**n = 35**	**n = 13**	**n = 22**		
	82.0 (70.0-95.0)	NA	72.0 (54.0-91.0)	NA	0.0386
rwDCR, %	**n = 35**	**n = 13**	**n = 22**		
	91.0 (82.0-100.0)	NA	86.0 (72.0-100.0)	NA	0.1638
rwDoR in months, median (range)	**n = 28**	**n = 12**	**n = 16**		
	5.9 (3.0-12.0)	5.9 (3.5-8.5)	5.5 (2.1-13.4)	NA	0.1474
	**n = 44**	**n = 18**	**n = 26**		
Median rwPFS, months (95% CI)	13.9 (7.9-14.9)	11.8 (7.3-27.0)	13.9 (6.1-15.9)	1.34 (0.6-3.0)	0.4590

rwPFS Rate at 24 months, % (95% CI)	15.0 (5.0-31.0)	32.0 (6.0-62.0)	10.0 (1.0-27.0)	NA	NA
	**n = 44**	**n = 18**	**n = 26**		
Median OS, months (95% CI)	25.3 (18.3-54.3)	NR (11.3-NA)	23.8 (17.7-26.1)	3.13 (1.2-8.4)	0.0236
OS Rate at 24 months, % (95% CI)	52.0 (36.0-66.0)	69.0 (41.0-86.0)	41.0 (21.0-59.0)	NA	NA
**3rd Generation**					
rwORR, %	**n = 141**	**n = 58**	**n = 83**		
	78.0 (71.0-84.0)	87.0 (79.0-96.0)	71.0 (61.0-80.0)	NA	0.0175
rwDCR, %	**n = 141**	**n = 58**	**n = 83**		
	95.0 (91.0-98.0)	96.0 (91.0-100.0)	93.0 (88.0-99.0)	NA	0.4884
rwDoR in months, median (range)	**n = 102**	**n = 46**	**n = 56**		
	6.9 (2.9-11.7)	7.2 (2.5-12.4)	6.5 (3.0-10.8)	NA	0.9144
	**n = 154**	**n = 63**	**n = 91**		
Median rwPFS, months (95% CI)	17.2 (13.2-23.3)	18.4 (14.6-NA)	17.0 (10.0-NA)	1.3 (0.8-2.2)	0.2794
rwPFS Rate at 24 months, % (95% CI)	37.0 (25.0-48.0)	38.0 (20.0-55.0)	37.0 (22.0-53.0)	NA	NA
	**n = 154**	**n = 63**	**n = 91**		
Median OS, months (95% CI)	31.2 (27.9-NA)	NR (20.8-NA)	29.6 (24.2-NA)	1.1 (0.6-2.3)	0.6917
OS Rate at 24 months, % (95% CI)	66.0 (54.0-76.0)	63.0 (42.0-78.0)	68.0 (53.0-79.0)	NA	NA

CI = confidence interval; mo = months; n = no. of patients; NR = not reached; OS = overall survival; rwDoR = duration of response; rwORR = real-world overall response rate; rwPFS = real-world progression-free survival; *TP53mt* = *TP53* co-mutation; *TP53wt* = *TP53* wild-type

EGFR mutation includes both exon 19 and exon 21 mutations.

Table S6. Efficacy Outcomes – Non-*EGFR*-TKI Monotherapy.

Variable	Overall	*TP53wt*	*TP53mt*	HR (95% CI)	*p* -value
rwORR, %	**n = 36**	**n = 12**	**n = 24**		
	80.0 (67.0-93.0)	75.0 (50.0-99.0)	83.0 (68.0-98.0)	NA	0.5515
rwDCR, %	**n = 36**	**n = 12**	**n = 24**		
	91.0 (82.0-100.0)	91.0 (76.0-100.0)	91.0 (80.0-100.0)	NA	1.0000
rwDoR in months, median (range)	**n = 26**	**n = 8**	**n = 18**		
	3.9 (1.9-8.0)	3.9 (2.1-6.5)	5.3 (1.8-10.8)	NA	0.8196
	**n = 89**	**n = 39**	**n = 50**		
Median rwPFS, months (95% CI)	8.0 (5.6-11.2)	10.0 (5.2-12.1)	7.4 (5.0-13.5)	0.9 (0.5-1.6)	0.6938
rwPFS Rate at 24 months, % (95% CI)	5.0 (1.0-16.0)	7.0 (0-28.0)	8.0 (1.0-23.0)		-
	**n = 89**	**n = 39**	**n = 50**		
Median OS, months (95% CI)	25.1 (18.1-38.6)	24.5 (14.7-NA)	25.6 (16.1-38.6)	1.3 (0.7-2.3)	0.4016
OS Rate at 24 months, % (95% CI)	52.0 (40.0-63.0)	50.0 (32.0-65.0)	54.0 (38.0-68.0)		-

CI = confidence interval; mo = months; n = no. of patients; rwDoR = duration of response; rwORR real-world overall response rate; OS = overall survival; rwPFS = real-world progression-free survival; *TP53mt* = *TP53* co-mutation; *TP53wt* = *TP53* wild-type

EGFR mutation includes both exon 19 and exon 21 mutations.

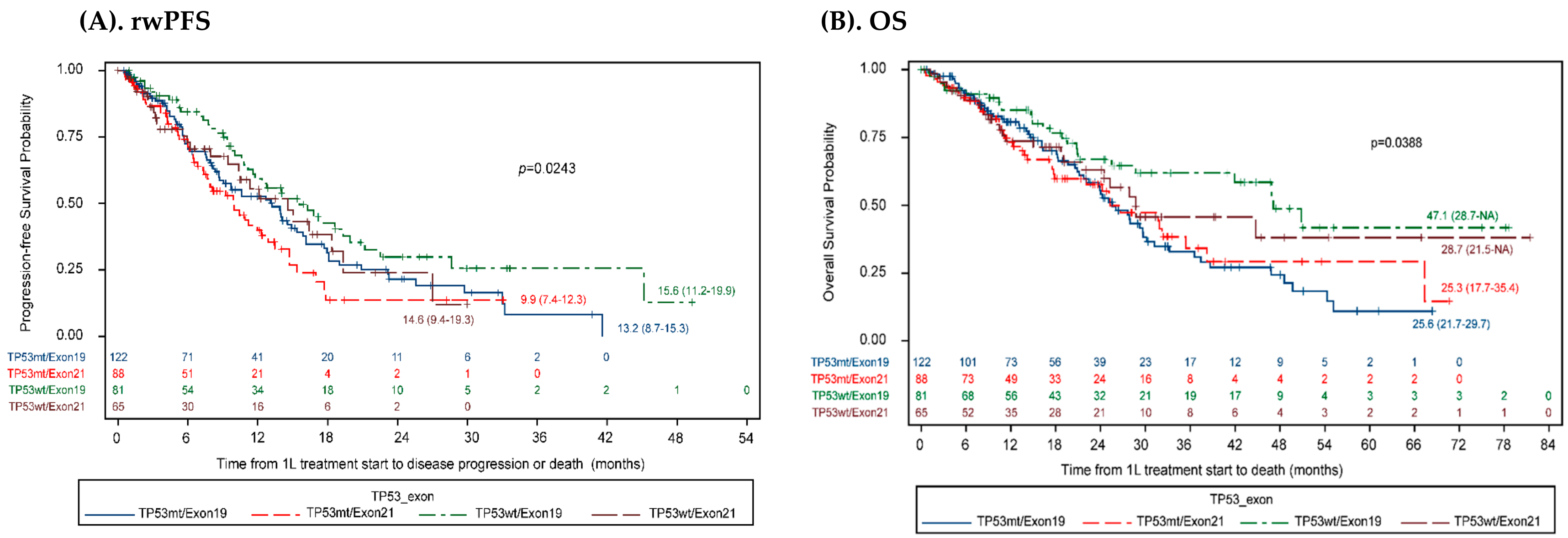

Figure S1. rwPFS and OS – *TP53 EGFR* Exon Groups.

CI = confidence interval; HR = hazard ratio; OS = overall survival; rwPFS = real-world progression free survival; *TP53mt* = *TP53* co-mutation; *TP53wt* = *TP53* wild-type exon 19 = exon 19 deletions, and exon 21 = exon 21L858R mutations.4-way comparison between the groups.
